# The Use of Fetal Noninvasive Electrocardiography

**DOI:** 10.1155/2016/5386595

**Published:** 2016-02-24

**Authors:** Igor Lakhno

**Affiliations:** Perinatology, Obstetrics and Gynecology Department, Kharkiv Medical Academy of Postgraduate Education, 58 Shalimov Street, Kharkiv 61176, Ukraine

## Abstract

Preeclampsia (PE) is one of the severe complications of pregnancy that leads to fetal deterioration. The aim was to survey the validity of fetal distress diagnostics in case of Doppler ultrasonic umbilical vein and arteries blood flow velocity investigation and ECG parameters analysis obtained from maternal abdominal signal before labor in preeclamptic patients. Fetal noninvasive ECG and umbilical arterial and venous Doppler investigation were performed in 120 patients at 34–40 weeks of gestation. And 30 of them had physiological gestation and were involved in Group I. In Group II 52 pregnant women with mild-moderate PE were observed. 38 patients with severe PE were monitored in Group III. The most considerable negative correlation was determined in pair Apgar score 1 versus T/QRS (*R* = −0.50; *p* < 0.05). So the increased T/QRS ratio was the most evident marker of fetal distress. Fetal noninvasive ECG showed sensitivity of 96.6% and specificity of 98.4% and, therefore, was determined as more accurate method for fetal monitoring.

## 1. A Due Diagnostic

Fetal monitoring is a main part of the antenatal surveillance system that could contribute to the improved perinatal outcome. The lack of accuracy of several fetal status investigation methods is well known [1, 2]. The main goal of fetal monitoring is duly diagnostics of fetal distress. Unfortunately, the introduction of most of instrumental techniques in perinatal medicine increased the level of obstetrical aggression. Typical concern is fetal growth restriction. The problem of immaturity is associated with unsubstantiated early delivery because of insufficient sensitivity and specificity of routine approaches to fetal compromise detection [2–6].

Fetal biophysical activity assessment methods are based on unborn baby neurobehavioral response investigation. These techniques require prolonged ultrasonic surveillance. Cardiotocography (CTG) demonstrates the sinus node response to the continual interaction of sympathetic and parasympathetic divisions of autonomic nervous system [4, 5]. The autonomic control of fetal cardiac rhythm could be investigated by fetal heart rate variability (HRV). CTG is a widely available method for fetal neurodevelopmental investigations based on cardiac rhythm reactivity to the fetal intrauterine motile activity in the antenatal period. The registration of the primary bioelectrical processes in the sinus node may be supposed to be more precious technique than mechanical detection of cardiac cycles used in CTG. But fetal noninvasive ECG has several evident problems now. The main one is low signal to noise ratio. Fetal noninvasive ECG extraction from maternal abdominal wall requires blind source separation and adaptive noise cancellation techniques application in the software [7–11]. Most of the fetal noninvasive ECG databases were obtained by magnetocardiographic registration. Such hardware is rather expensive. Ukrainian scientists developed “Cardiolab Babycard” fetal noninvasive ECG monitor [12]. The ECG recordings obtained in Ukraine were included in Physio Net database [13]. Fetal noninvasive ECG expectations are associated with the morphological parameters investigation additionally to the conventional HRV values. Fetal HRV parameters have a very wide range even in normal condition. The peculiarities of the fetal neurobehavioral response in active and sleepy periods may complicate the interpretation of conventional CTG tracing and increase the level of cesarean deliveries [5, 6, 10, 14]. It was revealed in several studies that elevated T-wave and increased T/QRS ratio were markers of fetal distress [9, 10, 14, 15]. These findings were due to *β*-adrenergic stimulation and anaerobic metabolic processes activation in fetal myocardium [10, 12]. The more recent investigations explored that fetal hypoxia could cause the shortening of QT interval [14].

Doppler ultrasonography is the most evident technological approach to the fetal well-being surveillance [2, 3]. The application of blood flow velocity parameters in the umbilical arteries and umbilical vein, ductus venosus, and aortic isthmus has a wide introduction in current perinatology. Increased maternal uterine arteries vascular resistance is known as a biophysical marker of preeclampsia (PE) [6]. Since PE is a prominent reason of fetal compromise it could be rather prospective to test the accuracy of Doppler ultrasonic methods and fetal noninvasive ECG in fetal distress diagnostics.

The investigation was aimed at surveying the validity of fetal distress diagnostics in case of Doppler ultrasonic umbilical vein and arteries blood flow velocity investigation and ECG parameters analysis obtained from maternal abdominal signal before labor in preeclamptic patients.

## 2. Materials and Methods

The study protocol was approved by the Bioethics Committee of the Kharkiv Medical Academy of Postgraduate Education. The eligible participants were informed about the methodology of the study, its aims, objectives, indications, and eventual complications before inclusion in the study. All patients who met the inclusion criteria gave written informed consent to participate in the investigation [16]. Inclusion criteria are as follows: diagnosed PE based on blood pressure more than 140/90 mm Hg on two separate occasions 6 hours apart and positive proteinuria test in two mild-stream urine samples collected 4 hours apart. Exclusion criteria are as follows: uterine contractions (threatened preterm labor), multiple pregnancy, eclampsia, and preexisting medical disorders like diabetes mellitus, cardiac diseases, renal disease, thyrotoxicosis, and chronic hypertension without superimposed PE.

120 patients at 34–40 weeks of gestation were enrolled in the study. And 30 of them had healthy pregnancy and were involved in Group I (control). 52 pregnant women with mild-moderate PE were observed in Group II. 38 patients with severe PE were monitored in Group III.

Doppler ultrasonography was performed on the ultrasound system “Voluson 730” (GE Healthcare, USA). Doppler tracing in umbilical arteries and vein was investigated. Maximum blood flow velocity (MBV) and pulsatile index (PI) in umbilical vein were studied. The obtained within 1 minute Doppler spectrogram of the venous umbilical blood flow was subjected to further processing. The curves of maximum blood flow velocity were isolated and their spectral components were determined. The spectra were calculated by sampling step Δ*t* = 0.01 seconds for a sample of 256 points. The resulting spectrum was obtained by averaging over all samples of this contingent [17].

Fetal ECG recordings were obtained from maternal abdominal signal with the application of fetal 5-channel noninvasive computer electrocardiographic system “Cardiolab Baby Card” (Scientific Research Center “KhAI-Medica,” Ukraine). The registration lasted for 10 minutes in the maternal sitting position. The values of total power (TP) and its spectral compounds, very low frequency (VLF), low frequency (LF), and high frequency (HF), were determined. The temporal characteristics of the fetal HRV, standard deviation of normal to normal intervals (SDNN), root mean square of successive heartbeat interval differences (RMSSD), the proportion of the number of pairs of NNs that differ by more than 50 ms divided by total number of NNs (pNN50), the amplitude of mode (the most frequent value of NN interval or the highest column in the histogram) − number of NN intervals included in the pocket corresponding to mode measured in percentage (%) (AMo), and stress index (SI) = AMo (%)/(2 × Mo × Var), Var = NN_max_ − NN_min_, were calculated. The morphological parameters of fetal ECG were investigated in average complex: duration of intervals (ms) QT and QRS, amplitude (mV) of the T-wave, and the ratio of T/QRS [18].

The share of instrumentally determined fetal distress was investigated in the antenatal period and Apgar score after delivery. The signs of fetal distress were absent or reversed diastolic blood flow in umbilical artery and pulsatile pattern of venous umbilical blood flow.

The results thus obtained were analyzed using the Chi-square test to compare for categorical data between the groups with the application of statistics software package Excel adapted for biomedical research. The significance was set at *p* value < 0.05. For the statistical analysis of relationship between *X* and *Y*, correlations coefficients were estimated using Spearman's test. Additionally, the sensitivity and specificity of diagnostic tests were calculated [19].

## 3. Results

The obtained results showed that umbilical venous blood flow velocity values were quite different in the study population (Table 1). The highest values of MBV and PI were determined in healthy pregnancy group. Further worsening of umbilical venous hemodynamics was revealed in Group II. The most considerable decrease of MBV and PI measured in Group III was associated with obvious pulsation of the venous wall.

**Figure 1:**
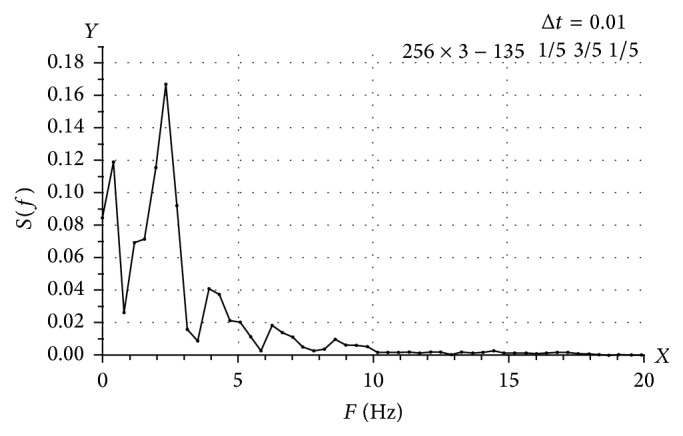
The spectral characteristics of the umbilical venous blood flow velocity in Group II.

The obtained spectral characteristics of the venous blood flow velocity explored the origin of the pacemakers in the study population. The umbilical venous hemodynamics was characterized with three mostly pronounced spectral peaks with frequencies values of 0.5 Hz, 2 Hz, and 7 Hz in Group I (Table 2). There was a considerable decrease in amplitude of the 7 Hz peak and even the absence of it in several cases in Group II (Figure 1). The most pronounced peak was recorded at a frequency of 2 Hz. And 0.5 Hz peak was lower. It was determined that two peaks in the region of 0.5 Hz and 7 Hz were almost absent in Group III. The pulsatile pattern of blood flow in severe preeclamptic patients was found. The amplitude of the only peak with frequency 2 Hz was rather high.

The fetal HRV parameters demonstrated decreased autonomic nervous regulation with abnormal relative predominance of sympathetic domain region values in PE groups (Table 3). The values of SDNN and TP were lower in Group II and Group III. But the shares of AMo, SI, and LF have been relatively elevated in total spectra of fetal HRV in preeclamptic patients. The decrease of RMSSD, pNN5O, and HF in patient with PE was determined in Group II and Group III. So it was found that there is the lack of vagal division of autonomic nervous regulation in PE. The gradual decline of VLF values in PE could reflect the decreased level of ergo- and trophotropic processes. The considerably decreased duration of QT interval in preeclamptic women was revealed. The elevation of T-wave led to the increased T/QRS ratio mostly in Group III.

**Figure 2:**
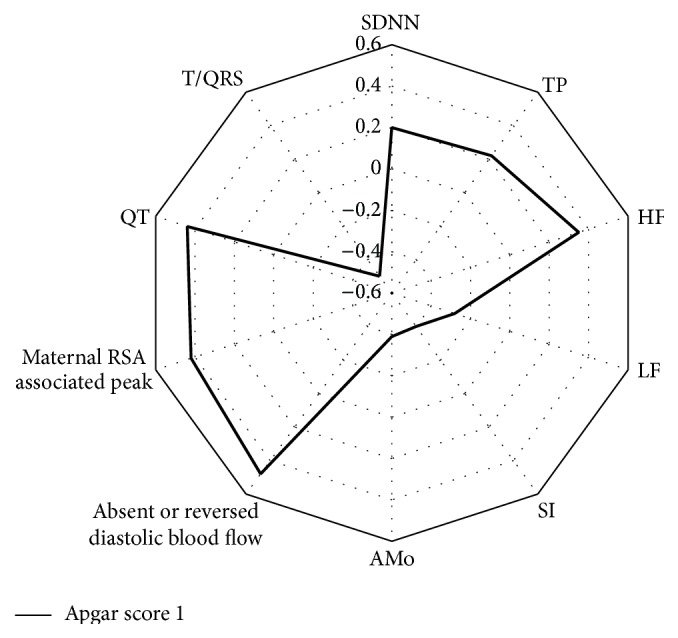
Correlations between Apgar score 1 and values of HRV, venous and arterial Doppler, and morphological ECG parameters in the study population.

The mean Apgar 1 values were 7.9 ± 0.8, 6.8 ± 0.7, and 6.2 ± 0.7, respectively, in Group I, Group II, and Group III. The investigation of statistically significant correlations between newborn Apgar score 1 and fetal HRV, arterial, and venous Doppler and noninvasive ECG parameters revealed certain regularities (Figure 2). The most considerable negative correlation was determined in pair Apgar score 1 versus T/QRS (*R* = −0.50; *p* < 0.05). So the increased T/QRS ratio was the most evident marker of fetal distress. The absent or reversed diastolic blood flow in the umbilical artery demonstrated correlation with Apgar score 1 (*R* = 0.48; *p* < 0.05). The QT interval duration revealed positive correlation with Apgar score 1 (*R* = 0.44; *p* < 0.05). It was found that the amplitude of 0.5 Hz associated peak had a certain correlation with Apgar score 1 (*R* = 0.42; *p* < 0.05). The validity of fetal HRV parameters was quite different. The negative correlation was determined between Apgar score 1 versus SI (*R* = −0.40; *p* < 0.05) and Apgar score 1 versus AMo (*R* = −0.39; *p* < 0.05). A considerable relationship in pairs was not determined: SDNN versus Apgar score 1 (*R* = 0.20; *p* < 0.05) and TP versus Apgar score 1 (*R* = 0.22; *p* < 0.05). It was confirmed that autonomic tone had a weak connection with fetal distress. The correlation between fetal vagal tone and Apgar score 1 was explored (*R* = 0.35; *p* < 0.05). The weak negative correlation between fetal sympathetic tone and Apgar score 1 was determined (*R* = −0.28; *p* < 0.05). The obtained data confirmed speculation that fetal noninvasive ECG parameters have considerable significance in fetal distress diagnostics.

## 4. Discussion

It was known that umbilical vein contracted slowly and the mean rate of vasomotion was 1.4 ± 0.05 cycles per minute [20]. Since three main oscillators in the umbilical hemodynamics were found the origin of the pacemakers became very relevant. And 2 Hz peak was possibly associated with arterial component of the blood flow. The sources of the two other prominent peaks were unclear. So the determined fluctuations with frequencies 0.5 Hz and 7 Hz captured the activity of the controlling signals in the system “mother-placenta-fetus.” The possible role of maternal factors in the regulation of the venous umbilical hemodynamics should be investigated later. It was possible to assume that above-mentioned oscillations contributed to the maintenance of continuous nonpulsatile type of umbilical venous blood flow. Maternal breathing was explored as a main factor of the fetal and maternal cardiac synchrony [21]. That is why respiratory sinus arrhythmia (RSA) could be considered as a possible driver of 0.5 Hz peak. Such frequency was not equal to maternal respiration rate. The speculation that the maternal RSA could play a trigger role and induce hemodynamic fluctuations capable of penetrating the placental site was performed. And the initial frequency could be changed in the process of penetration through placental barrier. But this thesis requires further investigations. It could be speculated that maternal RSA associated fluctuations were implicated in physiological mechanism of the fetal nutritional support [17]. The lack of maternal RSA mediated management of umbilical hemodynamics was found in patients with PE. Therefore, PE destroyed fetal and maternal hemodynamic coupling. It was found as a pathogenetic peculiarity of fetal distress in preeclamptic women. The revealed correlation between maternal RSA peak and Apgar 1 score confirmed the speculation.

It is well known that optimal uteroplacental hemodynamics is one of the main mechanisms of beneficial perinatal outcome. All catastrophic events such as preterm placental abruption could be associated with placental bed pathology [22]. So the function of uteroplacental hemodynamics is strongly depended on arterial inflow and venous outflow:

 
*V*_*p*_ = (*F*_*a*_ − *F*_*v*_)*dt*;  *dV*_*p*_ = (*F*_*a*_ − *F*_*v*_),
 
*V*_*p*_—the volume of blood in the placental circuit;
 
*F*_*a*_—arterial inflow;
 
*F*_*v*_—venous outflow.

As hemodynamical oscillations are obviously connected with maternal HRV it is possible to create such mathematical model of the uteroplacental hemodynamics:

 
*d* ^2^ *V*_*p*_/*dt* ^2^ = (*d*/*dt*)(*F*_*a*_ − *V*_*p*_);  *d* ^2^ *V*_*p*_/*dt* ^2^ + *w* ^2^ *V*_*p*_ = 0,
 
*V*_*p*_—the volume of blood in the placental circuit;
 
*F*_*a*_—arterial inflow;
 
*F*_*v*_—venous outflow;
 
*w*—natural frequency of the system.

However, this model does not describe the entropy (losses) in the system associated with an increased vascular resistance in PE. That is why the more realistic model could be formulated as *d* ^2^ *Vp*/*dt* ^2^ + (*d*/*dt*)(*Fa* − *Vp*) + *w* ^2^ *Vp* = 0. It is similar to van der Pol equation [23]. Maternal regulatory mechanisms provide uteroplacental hemodynamics. And maternal pacemakers probably could be considered as a natural frequency of the system in the above model.

Fetal hypersympatheticotonia was found as a typical pattern of neurodevelopmental response in preeclamptic women. But the lack of HRV parameters in fetal distress diagnostics was determined. The obtained data explored the use of SI and AMo as universal markers of fetal hypersympatheticotonia. It was determined that SI and AMo values were independent from fetal stationary condition in the antenatal period. But fetal noninvasive ECG morphological parameters demonstrated better validity as markers of fetal distress. The decreased QT duration and the rise of T-wave amplitude were the symptoms of *β*-adrenergic receptors activation and myocardial ischemic lesion [14]. Therefore, an increased T/QRS ratio was explored as a valid marker of fetal distress in several studies [7–12]. The presence of absent or reversed arterial diastolic component on Doppler blood flow tracing makes obstetrician think about fetal distress and urgent cesarean delivery. But sometimes additional time in case of fetal immaturity is necessary. The investigation of QT interval or T/QRS ratio could confirm or neglect this diagnosis and provide a doctor with extra time to accelerate fetal lung maturation. The question of the stability of Doppler ultrasonic and fetal noninvasive ECG signal tracings showed absolute (100.0%) result in Doppler successful registrations and a bit low (96.0%) rate of high precision ECG. Some additional measures to increase the quality of the fetal noninvasive ECG technique like dermabrasion should be provided by skilled staff.

The rate of fetal distress in patients with PE was 32.2%. The values of sensitivity and specificity of noninvasive fetal ECG were 96.6% and 98.4%, respectively. The absent or reversed arterial umbilical blood flow has showed sensitivity of 89.7% and specificity of 95.0%. The umbilical venous pulsatile blood flow has demonstrated sensitivity of 93.1% and specificity of 96.7%. These results were rather logical. The hemodynamic abnormalities in the system of “mother-placenta-fetus” are known as early determined features of fetal deterioration in PE [22, 24]. So fetal noninvasive ECG could provide more objective data based on fetal myocardial response to ischemia.

Fetal noninvasive ECG showed additional possibilities to conventional Doppler fetal monitoring. The confirmed accuracy may contribute to the reduction of obstetrical aggression.

## 5. Conclusion 

Fetal noninvasive ECG showed sensitivity of 96.6% and specificity of 98.4% and, therefore, was determined as more accurate method for fetal monitoring in PE.

## Figures and Tables

**Table 1:** The parameters of umbilical venous blood flow velocity in the study population.

Parameter, units of measure	Group I	Group II	Group III
MBV, cm/s	15.3 ± 4.8	13.2 ± 3.6^*∗*^	11.4 ± 3.2^*∗*/†^
PI, units	0.8 ± 0.2	0.6 ± 0.2^*∗*^	0.4 ± 0.1^*∗*/†^

Notes: *∗*, the differences were statistically significant compared to Group I (*p* < 0.05); †, the differences were statistically significant compared to Group II (*p* < 0.05).

**Table 2:** The amplitudes of the spectral peaks of umbilical blood flow velocity in the study population.

Frequency of peak	Group I	Group II	Group III
0.5 Hz, c.u.	0.14 ± 0.02	0.10 ± 0.02^*∗*^	0.03 ± 0.01^*∗*/†^
2 Hz, c.u.	0.16 ± 0.01	0.12 ± 0.01^*∗*^	0.18 ± 0.04^*∗*/†^
7 Hz, c.u.	0.15 ± 0.01	0.04 ± 0.01^*∗*^	0.03 ± 0.01^*∗*/†^

Notes: *∗*, the differences were statistically significant compared to Group I (*p* < 0.05); †, the differences were statistically significant compared to Group II (*p* < 0.05).

**Table 3:** Fetal HRV and morphological noninvasive ECG parameters in the study population.

Index	Group I	Group II	Group III
SDNN, ms	45.8 ± 13.1	29.4 ± 8.3^*∗*^	10.2 ± 4.5^*∗*/†^
RMSSD, ms	22.4 ± 3.4	14.2 ± 2.6^*∗*^	8.1 ± 0.8^*∗*/†^
pNN5O, %	8.7 ± 2.5	5.9 ± 1.8^*∗*^	2.0 ± 0.5^*∗*/†^
АМо, %	39.6 ± 14.1	50.2 ± 11.6^*∗*^	65.9 ± 13.4^*∗*/†^
SI, c.u.	169.3 ± 42.7	496.1 ± 65.8^*∗*^	1467.3 ± 405.8^*∗*/†^
TP, ms^2^	1513.6 ± 329.1	896.2 ± 163.5^*∗*^	424.9 ± 93.7^*∗*/†^
VLF, ms^2^	1252.8 ± 248.3	692.8 ± 91.3^*∗*^	251.8 ± 44.2^*∗*/†^
LF, ms^2^	184.3 ± 26.5	151.9 ± 34.1^*∗*^	135.0 ± 19.6^*∗*/†^
HF, ms^2^	77.6 ± 9.4	53.6 ± 8.2^*∗*^	38.9 ± 10.4^*∗*/†^
QT, ms	222.5 ± 28.6	209.2 ± 25.1^*∗*^	194.8 ± 31.3^*∗*/†^
QRS, ms	64.3 ± 11.8	64.6 ± 10.2^*∗*^	65.7 ± 11.9^*∗*/†^
T, mcV	2.8 ± 0.5	4.4 ± 1.6^*∗*^	8.2 ± 2.1^*∗*/†^
T/QRS	0.04 ± 0.01	0.08 ± 0.03^*∗*^	0.20 ± 0.05^*∗*/†^

Notes: *∗*, the differences were statistically significant compared to Group I (*p* < 0.05); †, the differences were statistically significant compared to Group II (*p* < 0.05).

SDNN: standard deviation of normal to normal intervals; RMSSD: root mean square of successive heartbeat interval differences; pNN50: the proportion of the number of pairs of NNs that differ by more than 50 ms divided by total number of NNs; AMo: the amplitude of mode (the most frequent value of NN interval or the highest column in the histogram) − number of NN intervals included in the pocket corresponding to mode measured in percentage (%); SI: stress index (SI) = AMo (%)/(2 × Mo × Var); Var = NN_max_−NN_min_; TP: total power; VLF: very low frequency; LF: low frequency; and HF: high frequency.
